# Adaptive Null Widening Beamforming Algorithm in Spatially Correlated Color Noise

**DOI:** 10.3390/s22166182

**Published:** 2022-08-18

**Authors:** Shijing Xiao, Bin Li, Qing Wang

**Affiliations:** National Key Laboratory of National Defense Technology for Integrated Ship Power Technology, Naval University of Engineering, Wuhan 430000, China

**Keywords:** spatially correlated color noise, robust adaptive beamforming, Toeplitz structure, projection transformation, linear constraint

## Abstract

Under the background of spatially correlated color noise, the incidence angle of a jamming signal in a high-speed moving platform rapidly changes, which leads to the degradation of the anti-interference performance and the waveform distortion of the adaptive beamformer. In this paper, a projection-constrained null broadening beamforming algorithm based on the Toeplitz matrix structure is proposed. The algorithm first extracts the subspace of the covariance matrix of the steering vector of the pre-determined extended angle interval and constructs the constraint matrix and the projection transformation matrix. The received signal covariance matrix with a Toeplitz structure is then constructed using the correlation number between each array element and the pre-set reference array element. Finally, the constructed covariance matrix is transformed through projection, and the weight of each array element is constrained by the constraint matrix. The theoretical optimal solution of adaptive wide null beamforming in spatially correlated color noise is obtained. The simulation results show that, compared with the existing robust adaptive beamforming algorithms, the proposed algorithm can efficiently improve the distortion of adaptive anti-jamming beams, and can achieve null broadening in the jamming direction under the condition of spatially correlated color noise, which improves the output signal to the interference-plus-noise ratio (SINR) of the adaptive beamformer.

## 1. Introduction

Adaptive beamforming technology is used in communication, radar, and navigation by adaptively adjusting the weights of array elements so that the beam forms nulls in the direction of the jammers, which efficiently suppresses the interference [1,2,3]. High-performance robust adaptive beamforming methods currently exist, such as the correlation matrix diagonal loading algorithm [4], eigenspace-based algorithm [5], and algorithm using semidefinite relaxation [6]. In an actual situation, the external environment background noise mainly contains ground clutter, sea clutter, atmospheric noise, solar noise, etc. This external environment background radiation is full of the whole three-dimensional space of continuous distributions of radiation sources. The array antennas in each array element facing the external environment are almost the same, and the external environment noise is correlated. The background noise of the array antenna is, therefore, often spatially correlated color noise [7,8,9,10]. On the other hand, the sample covariance matrix of the signal is obtained from the number of snapshots over a period of time. In addition, the finite number of snapshots results in the fact that the covariance matrix of the noise signal is not a diagonal matrix, the correlation between the array elements is non-ignorable, and the spatial correlation color noise characteristic of the background noise is further increased [11]. The color noise in the receiver leads to an increase in the distortion of the adaptive beamforming, such as a mainlobe shift and sidelobe elevation [12]. In addition, the adaptive array carries platforms, such as warplanes and missiles, which are often in a high-speed motion state. In this case, the rapid change in the position of the interferer source and the relatively slow update of the adaptive weights causes the interferer signal to not strictly fall in the narrow null of the adaptive beamforming. This results in the rapid degradation of the array’s anti-interference performance [13,14,15].

In the study of robust adaptive beamforming for color noise backgrounds, the performance of the modified covariance matrix-based beamforming algorithm depends on the accurate estimation of the noise covariance matrix, but the accurate estimation is very difficult to achieve [16,17,18]. The authors in [19] applied the Kalman filtering method for whitening the non-stationary colored noise, which is not applicable to the background of spatially correlated color noise. The reconstruction of interference-plus-noise covariance matrices is the current method for adaptive beamforming in color noise backgrounds with higher performance [20,21]. The literature [20] applies a co-prime array to estimate the direction-of-arrival (DOA) for each source by matching the super-resolution spatial spectra of a pair of sparse uniform linear subarrays and estimates the power of each source via joint covariance matrix optimization. In the literature [21], the entire airspace is divided into the desired signal airspace, the interference airspace, and the noisy signal airspace. In addition, the normalized cross-Capon power matrix is calculated in the delineated noisy airspace, and the colored noise covariance matrix (CNCM) can be further estimated. Combining the signal sample covariance matrix allows for the derivation of the expression for calculating the power of the interference. These covariance matrix reconstruction methods with complex calculation steps can lead to the slower updating of weights, so that these methods cannot be applied to scenarios where the array moves the platform at high speed, and the parameters selected with this algorithm affect the output performance of the array. The adaptive beamforming algorithm with a wide null is a robust beamforming algorithm that effectively solves the mismatch between the incident angle of the interference and the null angle of the adaptive beam. The covariance matrix sharpening can broaden the null, while the null depth becomes shallower and the array gain is reduced [22,23]. The wide null algorithm with derivative constraints [24,25] lets multiple null constraints spread the null in one direction, sacrificing large degrees of freedom, while the null width is not easily controlled. In recent years, the null broadening algorithm based on spatial projection [26] and linear constraint [27] has achieved null widening in a set area and controllable null depth. In these algorithms, the background noise of the array receiver is assumed to be Gaussian white noise, which results in an adaptive beam that is far less immune to interference than the simulated beam [28]. Therefore, a robust beam formation of high-speed motion array platforms in the background of spatially correlated color noise is crucial to guarantee the effective transmission of battlefield information.

This study is inspired by the fact that the rank of the received signal covariance matrix with a Toeplitz structure is not affected by the received signal correlation for solving the adaptive beamforming of coherent signals. The main contributions of this paper are threefold. Firstly, we design a robust adaptive beamforming algorithm, which considers the background of spatially correlated color noise in practical highly dynamic situations. Secondly, we analyze that the projection constraint allows the zero trap to be widened and deepened, and that the Toeplitz matrix structure can effectively reduce the effect of background color noise and derive the optimal weights for the array antenna. Thirdly, we simulate and compare the beamforming performance under spatially correlated color noise, and the anti-interference performance of different robust beamforming algorithms under colored noise and a high-speed motion platform. The simulation experiments show that the proposed algorithm can perform null broadening under spatially correlated color noise, while outputting a higher SINR and effectively suppressing beam distortion.

## 2. Wave Distortion in Spatially Correlated Color Noise

**Figure 1:**
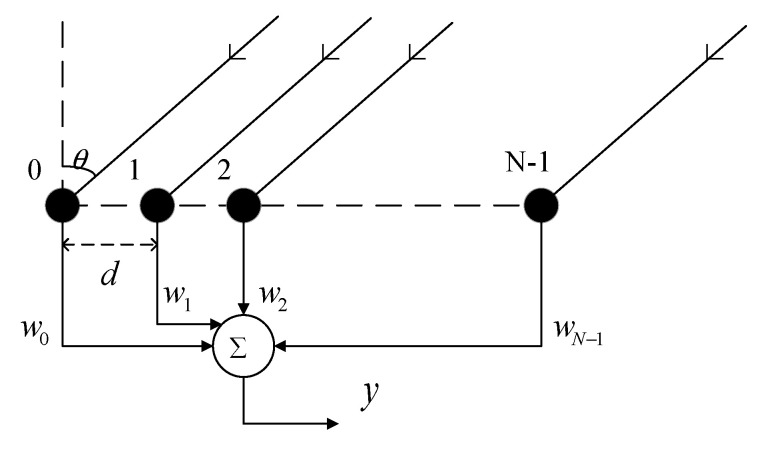
Uniform linear array.

From Figure 1, without the loss of generality, a uniform linear array (ULA) with N element spacing of d was considered. There was one non-circular desired signal from $\theta_{0}$ and P non-circular interferences from $\theta_{k}(k=1,2,\cdots ,P)$ incident to the array, whose envelops were $s_{0}(t)$ and $s_{k}(t)(k=1,2,\cdots ,P)$, respectively.

The signal $x_{l}(t)(l=1,2,\cdots ,N)$ received by the l element in the array could then be expressed as [29]:

(1) $$x_{l}(t)=a(\theta_{s})s_{0}(t)+\sum_{k=1}^{P}a(\theta_{k})s_{k}(t)+n_{l}(t)$$

where the steering vector of a uniform linear array is given by:

(2) $$a(\theta )={\left[1,e^{-j2\pi d\sin\theta /\lambda },\cdots ,e^{-j2\pi (N-1)d\sin\theta /\lambda }\right]}^{T}$$

where [.]^T^ stands for the transpose, the envelope of the non-circular signal has the character of $s(t)=s^{*}(t)$, []^*^ stands for the conjugate, λ is the wavelength, $A=[a(\theta_{0}),a(\theta_{1}),\ldots ,a(\theta_{P})]$ is the steering vector matrix of the array, θ is the signal incident angle relative to the array, and $n_{l}(t)$ is the noise received by the l element in the array.

Using Equation (1), the covariance matrix $R_{X}$ of the received signal was given by:

(3) $$R_{X}=E[X(t)X^{H}(t)]=AR_{S}A^{H}+R_{n}$$

where $X(t)={\left[x_{1}(t),x_{2}(t),\cdots ,x_{M}(t)\right]}^{T}$ is the signal vector received by the uniform linear array, $R_{S}=E[s(t)s^{H}(t)]$ is the covariance matrix between the interference and the desired signal, $s(t)={\left[s_{0}(t),s_{1}(t),\cdots ,s_{P}(t)\right]}^{T}$ is the envelope of the desired signal and the interference received by the uniform linear array, $R_{n}=E[N(t)N^{H}(t)]$ is the correlation matrix of the noise signal, and $N(t)={\left[n_{1}(t),n_{2}(t),\cdots ,n_{M}(t)\right]}^{T}$ is the noise signal vector received by the uniform linear array. When the background noise was Gaussian white noise, the covariance matrix of the noise signal $R_{n}=\sigma_{n}^{2}I$ was a diagonal matrix, where $\sigma_{n}^{2}$ is the noise power of a single array element. However, in the background of color noise, the covariance matrix of the noise signal was not a diagonal matrix. In the sequel, the influence of the spatially correlated color noise on the adaptive beamforming was analyzed in the presence of a single interference in space.

Usually, the power of the desired signal received by the array is much less than the power of the interference. At this time, the covariance matrix of the received signal could be approximated using the covariance matrix of the interference and noise as:

(4) $$R_{X}\approx \pi_{J}a(\theta_{j})a^{H}(\theta_{j})+R_{n}$$

where $a(\theta_{j})$ is the steering vector of the interference and $\pi_{J}$ is the power of interference.

According to the maximum signal-to-noise ratio criterion, the adaptive weight of the linear array was given by:

(5) $$w=\mu R_{X}^{-1}a(\theta_{s})$$

where $a(\theta_{s})$ is the steering vector of the desired signal. In addition, μ could be expressed as:

(6) $$\mu =\frac{1}{a^{H}(\theta_{s})R_{{}^{X}}^{-1}a(\theta_{s})}$$

The inverse of Equation (4) was then obtained:

(7) $$R_{X}^{-1}=R_{n}^{-1}-\frac{\pi_{J}R_{n}^{-1}a(\theta_{J})a^{H}(\theta_{J})R_{n}^{-1}}{1+\pi_{J}a^{H}(\theta_{J})R_{n}^{-1}a(\theta_{J})}$$

When the interference power was large, Equation (7) could be substituted into Equation (5) to obtain:

(8) $$w=\mu (I-\frac{R_{n}^{-1}a(\theta_{J})a^{H}(\theta_{J})}{a^{H}(\theta_{J})R_{n}^{-1}a(\theta_{J})})R_{n}^{-1}a(\theta_{s})$$

The adaptive beam pattern was expressed as:

(9) $$B(\theta )=\left|w^{H}a(\theta )\right|$$

where a(θ) is the steering vector with an incident angle of θ in the uniform linear array. 

In the case of white noise, the weight of the array $w_{1}$ and the directional gain of the adaptive beam pattern $B_{1}(\theta )$ could be obtained by combining the above equations:

(10) $$w_{1}=\mu^{\prime }(I-\frac{a(\theta_{J})a^{H}(\theta_{J})}{a^{H}(\theta_{J})a(\theta_{J})})a(\theta_{s})$$

and:

(11) $$B_{1}(\theta )={\left|\mu^{\prime }a^{H}(\theta_{s})(I-\frac{a(\theta_{J})a^{H}(\theta_{J})}{a^{H}(\theta_{J})a(\theta_{J})})a(\theta )\right|}^{2}$$

where $\mu^{\prime }=\sigma_{n}^{2}\mu$. The adaptive weights in the white noise environment were orthogonal to the interference vector, thus, creating a null in the interference direction, and the beam shape was approximately similar to the static directional map in the other directions. In the background of color noise, the weights of the array $w_{2}$ and the adaptive beam pattern $B_{2}(\theta )$ were, respectively:

(12) $$w_{2}=\mu (I-\frac{R_{n}^{-1}a(\theta_{J})a^{H}(\theta_{J})}{a^{H}(\theta_{J})R_{n}^{-1}a(\theta_{J})})R_{n}^{-1}a(\theta_{s})$$

and:

(13) $$B_{2}(\theta )={\left|\mu^{\prime }a^{H}(\theta_{s}){\left(R_{n}^{-1}\right)}^{H}(I-\frac{R_{n}^{-1}a(\theta_{J})a^{H}(\theta_{J})}{a^{H}(\theta_{J})R_{n}^{-1}a(\theta_{J})})a(\theta )\right|}^{2}$$

An oblique projection of the adaptive array with a weight of $R_{n}^{-1}a(\theta_{s})$ could then be performed in the spatially correlated color noise environment. Therefore, in the spatially correlated color noise environment, since the adaptive weight was still orthogonal to the direction of the interference, the null could be formed in the interference direction. In other directions, because the noise correlation matrix $R_{n}$ was not a diagonal matrix, different weights could be applied in the other directions, which resulted in different adaptive beams and static patterns, resulting in waveform distortions.

## 3. The Proposed Algorithm

In this section, a robust beamforming algorithm based on the construction of the projection transformation and constraint matrix, the received signal covariance matrix with a Toeplitz structure, and the calculation of the optimal array weight was proposed. It effectively solved the problem of robust beamforming of a high-speed motion platform array under the condition of spatially correlated color noise.

### 3.1. Construction of the Projection Transformation and Constraint Matrix

Through prior estimation, the signal angle interval to be widened was Θ, and the steering vector matrix between the interval was expressed as [30,31]:

(14) $$R_{\theta }=\int_{\Theta }\bar{a}(\theta ){\bar{a}}^{H}(\theta )d\theta$$

where (.)^H^ denotes the Hermitian transpose operator.

Since $R_{\theta }$ was a Hermite matrix, it was decomposed and the eigenvectors $u_{1},u_{2},\cdots ,u_{L}$ corresponding to L large eigenvalues were used as the basis vector to form the eigen-subspace $U_{L}$ of $R_{\theta }$ as:

(15) $$U_{L}=span\{u_{1},u_{2},\cdots ,u_{L}\}$$

In addition, the matrix P which was composed of the eigenvectors $u_{1},u_{2},\cdots ,u_{L}$ could be expressed as:

(16) $$P=[u_{1},u_{2},\cdots ,u_{L}]$$

The basis vector in $U_{L}$ was constructed as a projection operator:

(17) $$T=\sum_{k=1}^{L}u_{k}u_{k}^{H}$$

The gain in the desired signal direction was constant, and the weight vector of the array w was orthogonal to the subspace $U_{L}$ to ensure the null broadening in the pre-determined region Θ. A constraint equation then existed:

(18) $$w^{H}\left[a(\theta_{S}),P\right]=\left[1,0\right]$$

Afterwards, the constraint matrix C and the constrained response matrix were computed as:

(19) $$C=\left[a(\theta_{S}),P\right]$$

(20) $$f=[1,\underset{M}{\underset{︸}{0,\cdots ,0}}{]}_{1\times (M+1)}$$

The noise component in the signal was further reduced through a projection transformation, and the null depth was further deepened due to the power limitation in the widened region.

### 3.2. The Received Signal Covariance Matrix with Toeplitz Structure

The received signal covariance matrix with a Toeplitz structure was proposed for the conventional covariance matrix of coherent sources with insufficient rank. The rank of the correlation matrix of the Toeplitz structure was only related to the incident angle of the signal, and it was not affected by the signal correlation. Using the constructed matrix with a Toeplitz structure to replace the covariance matrix of the received signal effectively reduced the influence of the correlation of the received noise of each array element on beamforming.

Using the first array element in the linear array as the reference array element, the correlation coefficient between this array element and the received data of an array element in the array was given by [32]:

(21) $$\begin{array}{ll} r_{1l} & =E[x_{1}(t)x_{l}^{H}(t)]\\ & =A(1)R_{S_{0}}A^{H}(k)+R_{n}(1,k) \end{array}$$

where k=1,2,⋯,M, $x_{1}(t)$ is the signal received by the first array element, $x_{l}(t)$ is the signal received by the k element in the array, A(k) is the kth row of the steering vector matrix, $R_{S_{0}}$ represents the correlation matrix of the received desired signal, and $R_{n}(1,k)$ is the data in the first row and k column of the covariance matrix of spatially correlated color noise in the array. By constructing the Toeplitz matrix structure, the covariance matrix of the received signal was replaced with:

(22) $$R_{T}=\left[\begin{array}{cccc} r_{11} & r_{12} & & r_{1M}\\ r_{12}^{*} & r_{11} & & r_{1,M-1}\\ & & \ddots & \\ r_{1M}^{*} & r_{1,M-1}^{*} & & r_{11} \end{array}\right]$$

### 3.3. Calculation of the Optimal Array Weight

The projection transformation of the correlation matrix was expressed as:

(23) $${\bar{R}}_{T}=TR_{T}T^{H}$$

The proposed algorithm performed a projection transformation on the received signal, which enhanced the signal subspace and improved the orthogonality between the signal subspace and the noise subspace. Such an approach further reduced the height of the sidelobe. It can be seen in the simulation that the mainlobe width was slightly widened. According to the criterion of the maximum signal to the interference-plus-noise ratio, the Lagrange function for calculating the weight was constructed as:

(24) $$L(w)=w^{H}{\bar{R}}_{T}w+\lambda (w^{H}C-f)$$

By solving Equation (24), it was deduced that the optimal weight of the array null broadening under the background of spatially correlated color noise was given by:

(25) $$w_{opt}={\bar{R}}_{T}^{-1}C{\left(C^{H}{\bar{R}}_{T}^{-1}C\right)}^{-1}f^{H}$$

## 4. Simulation and Performance Analysis

In this paper, the simulation was built on a uniform linear array with 10 elements and half-wavelength spacing between adjacent elements. The desired signal was incident to the array from 0 degrees, two independent interferences were incident to the array from 30 degrees and −30 degrees, and the interference-to-noise ratio was 30 dB. The color noise in the simulation was spatially correlated color noise, and the covariance matrix of the color noise in the array was $R_{n}(a,b)=\sigma_{n}^{2}0.7^{\left|a-b\right|}e^{j\pi (a-b)/10}$, where $\sigma_{n}^{2}$ is the noise power. To analyze the performance of the proposed Toeplitz null broadening (TNB) algorithm, it was compared with the covariance matrix reconstruction of interference-plus-noise (RINC) [21], the variable diagonal loading (VLD) [33], the maximum eigenspace-based (LESB) algorithm [34], and the classical sample matrix inversion (SMI) [35]. The diagonal loading factor $\gamma =10\sigma_{n}^{4}$ in Equation (10) in [33] and the noise incidence angles in [21] were set to −85, −66, −47, −25, −9, 9, 25, 47, 66, and 85 degrees. It was assumed that due to the rapid movement of the platform, the direction of the incidence of the disturbance may have deviated from that which occurred by plus or minus three degrees. In addition, the null spreading angle interval of the proposed algorithm was [–33, 27] degrees and [27, 33] degrees, and the number of subspace basis vectors was five. All the experimental results were obtained from 100 independent Monte Carlo experiments.

### 4.1. Performance Analysis of the Proposed Algorithm under Different Background Noises

**Figure 2:**
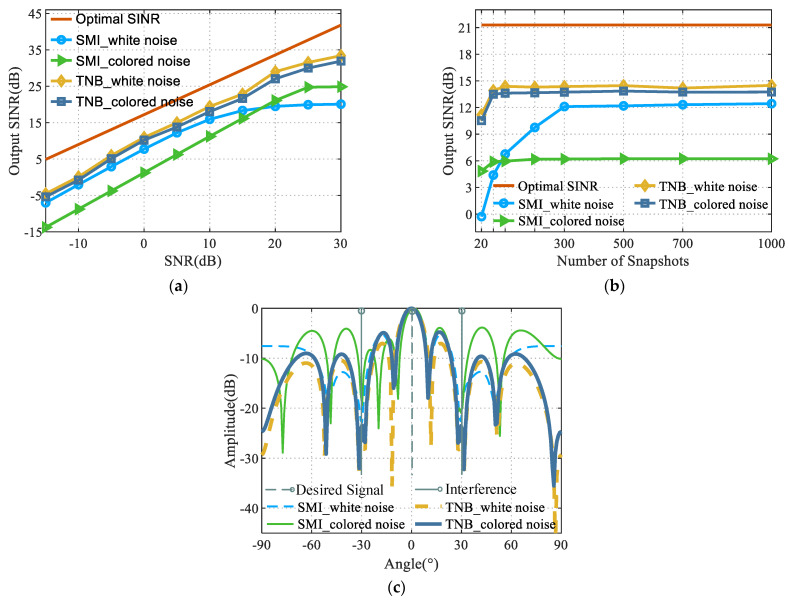
Anti-interference performance of SMI and the proposed algorithm under different background noises. (**a**) Performance comparison under different SNR values. (**b**) Performance comparison under different snapshot numbers. (**c**) Anti-jamming beam pattern of the SMI and the proposed algorithm under different background noises.

This section analyzed the anti-interference performance of the SMI algorithm and the proposed algorithm under the background of white noise and spatially correlated color noise, respectively. The obtained results are shown in Figure 2. More precisely, Figure 2a shows the variation curves of the output SINR with the input signal-to-noise ratio (SNR) for the two algorithms under different background noises for a snapshot number of 2000. Figure 2b shows the variation curves of the output SINR of the two algorithms at different snapshot numbers for an input SNR of 5 dB. Figure 2c shows the anti-interference beam formed with the array using the two algorithms for a number of snapshots of 2000 and an input SNR of 5 dB.

A comparative analysis of Figure 2 led to the following conclusions:

It can be seen from Figure 2a that the SMI and TNB algorithms outputted higher SINR in the white noise background, compared with the spatially correlated color noise background. The background condition of spatially correlated color noise reduced the anti-interference performance of the array adaptive beam. On the contrary, the output SINR of the proposed algorithm in the white noise and color noise backgrounds was similar, and the background noise had less influence on the anti-interference performance of the proposed algorithm.

The SMI algorithm increased the output SINR, while the SNR ratio gradually increased. When the input SNR continued to increase, the SMI algorithm appeared to be self-canceling, and the output SINR decreased as the SNR increased. On the contrary, in this experiment, the proposed algorithm did not show an inflection point of the degradation of anti-interference performance when the SNR increased.

It can be seen from Figure 2b that the proposed algorithm needed almost 60 snapshots to converge to the maximum output SINR with a constant input signal-to-noise ratio. On the contrary, the SMI algorithm was greatly affected by background noise, and it took 300 fast beats to converge to the maximum output SINR in the white noise background, while the maximum output SINR could be reached in 60 fast beats in the color noise background. However, the output SINR after convergence in the color noise background was lower.

It can be seen from Figure 2c that the sidelobe of the anti-interference beam map of the proposed algorithm in the color noise background was slightly higher than that in the beam map in the white noise background. In addition, the null depth and null width were mainly the same. On the contrary, the sidelobe of the SMI algorithm in the color noise background significantly increased, and the mainlobe was shifted by a certain angle.

In summary, the proposed algorithm was more adaptable to different background noises and had a stable interference suppression under different background noises.

### 4.2. Performance Analysis of the Algorithms under Spatially Correlated Color Noise

**Figure 3:**
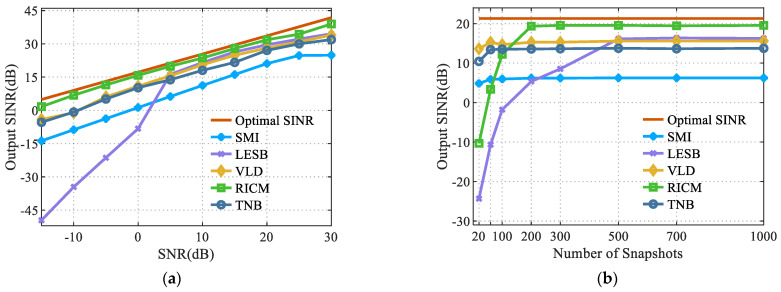

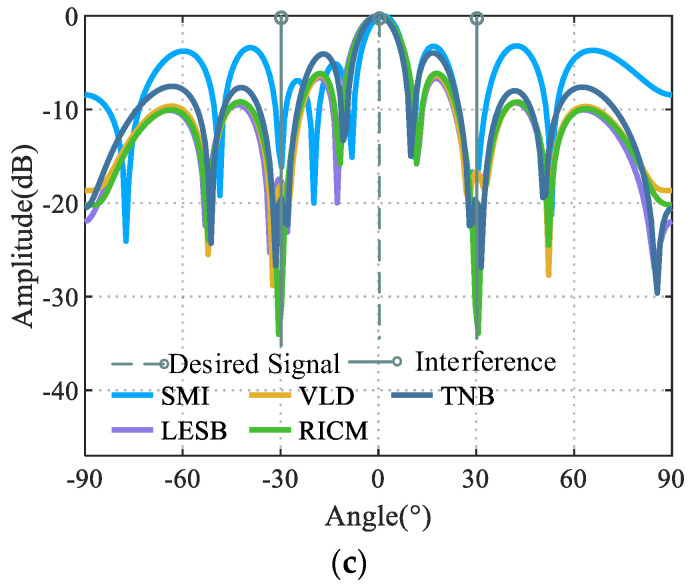
Anti-interference performance of different algorithms under spatially correlated color background noise. (**a**) Performance comparison under different SNR values. (**b**) Performance comparison under different snapshot numbers. (**c**) Anti-jamming beam pattern of the algorithms under different background noise.

This section analyzed the performance of different robust beamforming algorithms under spatially correlated color noise. The obtained results are shown in Figure 3. More precisely, Figure 3a shows the variation curves of the algorithm’s output SINR at different input SNRs for a number of snapshots of 2000. Figure 3b shows the variation curves of the algorithm’s output SINR at different snapshot numbers for an input SNR of 5 dB. Figure 3c shows the anti-interference beam of the algorithm at a snapshot number of 2000 and an input SNR ratio of 5 dB.

A comparative analysis of Figure 3 led to the following conclusions:

It can be seen from Figure 3a that, when the number of snapshots was large enough, the proposed algorithm had an output SINR similar to that of other robust beamforming algorithms under different signal-to-noise ratios. The SMI algorithm outputted the lowest SINR under the same conditions, while the LESB, VLD, and the proposed algorithms outputted a large SINR, which was almost 7 dB greater than that of the SMI algorithm. However, the output SINR and the interference suppression effect of the LESB algorithm rapidly decreased when the input SNR was low, and, therefore, it was not suitable for the case of low input SNR.

According to the analysis of Figure 3a,b, the output SINR of the proposed algorithm was less than that of the RICM algorithm by almost 5 dB. When the input SNR of the adaptive filter was the same, the number of snapshots required for the SMI, VLD, and the proposed algorithms to converge to the maximum output SINR was almost 60, while that required for RICM to converge to the maximum output signal-to-interference noise ratio was almost 200. The number of snapshots required for the proposed algorithm to converge to the maximum output SINR was low. The RICM algorithm estimated the normalized cross-Capon power matrix in the divided noisy area and calculated the covariance of the noise signal and the covariance of the interference signal relatively accurately. Compared with the proposed algorithm, the influence of spatial correlation colored noise was reduced by constructing the Toeplitz matrix. The RICM algorithm was closer to the beamforming criterion of the maximum output SINR and could reduce the correlation of the spatially correlated color noise more effectively. At the same time, the complexity of the algorithm was also higher. It could be seen from Figure 3c that under the background of spatially correlated color noise, the beam pattern of the RICM algorithm had the deepest null depth. The null depth formed with the proposed algorithm was similar to that of VLD and LESB, and the sidelobe was slightly higher than that of the VLD, LESB, and RICM algorithms.

The proposed, LESB, and VLD algorithms had a similar SINR under the background of color noise. Under the condition of sufficient snapshots, the interference suppression effect of the proposed algorithm was not as good as that of the RICM algorithm. However, when this algorithm converged to the maximum SINR, the required number of snapshots was small, and the RICM algorithm had many steps. The selection of the input angle of the analog noise signal was uncertain, which resulted in the instability of the anti-interference performance. In conclusion, the proposed algorithm had a stronger engineering adaptability.

### 4.3. Performance Analysis of the Algorithms in the Case of Angle Mismatch

**Figure 4:**
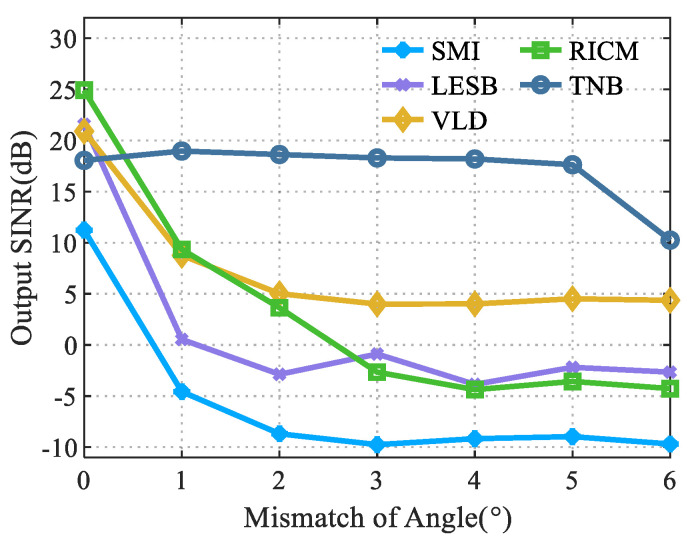
Anti-interference performances of different algorithms in angle mismatch.

This section simulated the anti-jamming performance of different robust beamforming algorithms in the case of an angle mismatch between the jamming signal angle and the null angle of the adaptive beamforming in the case of a spatially correlated color noise. Figure 4 shows the output SINR of each algorithm at different angle mismatches.

It can be seen from Figure 4 that the proposed algorithm had high anti-interference performance when the mismatch angle was less than 5 degrees. When the angle mismatch was 6 degrees, the anti-interference performance of the algorithm rapidly decreased because it was out of the range of null broadening. Compared with the proposed algorithm, due to the narrow nulling of the formed beam, when the angle was mismatched, the output SINR rapidly decreased. After the mismatching angle was 3 degrees, the output SINR tended to be stable with a slight change. Compared with the RICM algorithm which had the highest performance, when the interference angle and null angle were matched, the output SINR of the proposed algorithm was almost 7 dB higher after the mismatch convergence. The anti-interference performance of the proposed algorithm was basically unaffected in the null broadening range when the mismatch occurred, which was almost 14 dB higher than that of the RICM algorithm. Compared with the VLD algorithm, which had the highest anti-jamming performance in the case of mismatches, the output SINR was almost 6 dB higher. In summary, the proposed algorithm had a more stable anti-interference performance under the background of spatially correlated color noise and a high-speed moving array platform.

## 5. Conclusions

In this paper, the problem of robust beamforming for a high-speed moving array platform under the background of spatially correlated colored noise was studied. After analyzing the influence of the background noise on the adaptive beamforming, an adaptive wide null beamforming algorithm based on the Toeplitz matrix structure projection constraint was proposed.

The subspace of the integration of the correlation matrix of the steering vector in the pre-determined extended region was first extracted, and the constraint matrix and the projection transformation matrix were constructed. The covariance matrix of the array-received signal with a Toeplitz structure was then constructed using the correlation numbers of the received data of each array element and the reference array element. Finally, the optimal weight vector of the array was obtained through a projection transformation and linear constraint on the constructed covariance matrix. The simulation results showed that the anti-jamming performance of the proposed algorithm was less affected by the background noise. It could perform the null broadening of the beam in the pre-determined region and solve the problems of distortion sidelobe lifting and mainlobe offset of adaptive beamforming caused by spatially correlated color noise.

## Data Availability

Not applicable.
